# Assessing professionalism in mental health clinicians: development and validation of a situational judgement test

**DOI:** 10.1192/bjo.2023.582

**Published:** 2023-11-13

**Authors:** Lauren M. E. Aylott, Gabrielle M. Finn, Paul A. Tiffin

**Affiliations:** Health Professions Education Unit, Hull York Medical School, University of York, UK; Division of Medical Education, School of Medical Sciences, University of Manchester, UK; and Health Professions Education Unit, Hull York Medical School, University of York, UK; Health Professions Education Unit, Hull York Medical School, University of York, UK; and Department of Health Sciences, University of York, UK

**Keywords:** Mental health services, personnel selection, professionalism, situational judgement testing, procedural knowledge

## Abstract

**Background:**

Situational judgement test (SJT) scores have been observed to predict actual workplace performance. They are commonly used to assess non-academic attributes as part of selection into many healthcare roles. However, no validated SJT yet exists for recruiting into mental health services.

**Aims:**

To develop and validate an SJT that can evaluate procedural knowledge of professionalism in applicants to clinical roles in mental health services.

**Method:**

SJT item content was generated through interviews and focus groups with 56 professionals, patients and carers related to a large National Health Service mental health trust in England. These subject matter experts informed the content of the final items for the SJT. The SJT was completed by 73 registered nurses and 36 allied health professionals (AHPs). The primary outcome measure was supervisor ratings of professionalism and effectiveness on a relative percentile rating scale and was present for 69 of the participating nurses and AHPs. Personality assessment scores were reported as a secondary outcome.

**Results:**

SJT scores statistically significantly predicted ratings of professionalism (β = 0.31, *P* = 0.01) and effectiveness (β = 0.32, *P* = 0.01). The scores demonstrated statistically significant incremental predictive validity over the personality assessment scores for predicting supervisor ratings of professionalism (β = 0.26, *P* = 0.03).

**Conclusions:**

These findings demonstrate that a carefully designed SJT can validly assess important personal attributes in clinicians working in mental health services. Such assessments are likely to represent evidence based, cost-effective tools that can support values-based recruitment to mental health service roles.

‘Values guide the selection or evaluation of actions, policies, people and events. People decide what is good or bad, justified or illegitimate, worth doing or avoiding, based on possible consequences for their cherished values.'^1^ Values-based recruitment (VBR) was introduced in England in 2014 to ensure that students, trainees and employees of healthcare services are selected on the basis that their values and behaviours align with the values stated in the National Health Service (NHS) constitution.^2^ A significant driver of VBR was the findings of the Francis report, which documented the failings of Mid Staffordshire NHS Foundation Trust. It is particularly important that staff exhibit the right values and behaviours in mental health and learning disability settings where clients are more vulnerable to abuse and exploitation.^3^ The care scandal uncovered at Winterbourne View is a tragic example of what can occur when this is not the case.^4^ Moreover, prior research has found that lapses of ‘fitness to practise’ in medicine are far more often related to personal conduct as opposed to clinical competence.^5^ Thus, there is international interest in defining and selecting personnel in relation to appropriate values, related attitudes and behaviours for work in all healthcare settings.^6,7^ However, operationalising and measuring such constructs has been challenging in practice.

## Assessment of non-academic attributes

Various approaches have been used for the selection of healthcare staff in an attempt to evaluate the extent to which appropriate values and attitudes are understood, held and exhibited. These have included the use of personal references, structured interviews and personality tests.^8^ However, many experts have advocated the use of situational judgement tests (SJTs) as a potentially valid, cost-effective assessment method that can be used as a component of personnel selection to medical and other healthcare roles.^9,10^ SJTs have been referred to as ‘low fidelity simulations’.^11^ Test takers are given hypothetical work-related scenarios and are subsequently asked to exercise their judgement, by evaluating alternative courses of action (an example of an SJT item is shown in Box 1). Meta-analytic studies report that, in general, SJT scores predict interpersonal aspects of actual job performance, providing evidence of criterion-related validity.^12^ This is also true of SJTs used in medical selection.^13^ The scores from SJTs used in personnel selection have been observed to often correlate with various personality traits, including emotional stability, conscientiousness and agreeableness.^14^ Furthermore, in some cases, SJTs have been found to provide incremental validity over and above that related to measures of cognitive ability and personality traits when predicting job performance.^15,16^ The tests can be delivered digitally and at scale, often making them cost-effective alternatives to more resource-intensive approaches such as face-to-face interviews. As yet, however, no SJT has been developed and validated specifically for use in a mental health services context.

Box 1 Example item on a situational judgement test for selection into mental health services

SCENARIO. You work in a community mental health team. You attend a hospital discharge meeting for one of your patients. You disagree with the care plan being put forward by the ward staff, as they want you to see the patient three times a week. You know, given your current caseload, that it is not possible to sustain this level of support for the patient. The patient and their family are also present at the meeting.

*How **appropriate** would it be to respond in the following manner?*

To agree, in front of the patient, that you will fulfil the care plan and you will visit the patient three times a week.

- Very appropriate
- Appropriate, but not ideal
- Inappropriate, but not awful
- Very inappropriate

## Research aims and hypotheses

For the reasons stated above, the current study sought to develop and validate an SJT to assess an individual's procedural knowledge of professionalism for mental health services. Such knowledge is important for manifesting behaviours congruent with desirable values when delivering mental healthcare. Thus, such an SJT would potentially support VBR in this context. It was hypothesised that scores on the SJT would be related to supervisory ratings of perceived *professionalism* and *effectiveness*. In addition, we sought to explore whether SJT scores provided incremental validity over personality assessment ratings.

## Method

### Ethics statement

The authors assert that all procedures contributing to this work comply with the ethical standards of the relevant national and institutional committees on human experimentation and with the Helsinki Declaration of 1975, as revised in 2008. All procedures involving human subjects/patients were approved by the relevant committees. The qualitative study used to develop the SJT content received a favourable ethical opinion from London – Camden & Kings Cross Research Ethics Committee (REC reference: 18/LO/0630), the Health Research Authority and the University of York Health Sciences Research Governance Committee. The validation study, as NHS staff-based research, received approval from the Health Research Authority (19/HRA/6403) and Hull York Medical School Ethical Committee. All participants provided written informed consent to participate.

### Development of the SJT

An initial operational definition of professionalism for a mental health services context was derived from a previous systematic review; the authors coined the term ‘working professionalism’. This referred to mental health practitioners’ ‘ability to form judgements and act accordingly, thinking critically and using reflection in action’.^17^ That is, professionals must possess ‘practical wisdom’ if they are to work effectively in mental health services. In this sense, ‘practical wisdom’ refers to an ability to apply values flexibly, appropriately and effectively in a situation-specific manner. This could be conceptualised as ‘tacit knowledge’, something previously evaluated using SJT-type assessments.^18^ The initial pool of SJT items was developed from data collected during interviews and focus groups with patients, carers and professionals working in mental health services (*n* = 56; see refs ^19,20^); interviews and focus groups focused on the concept of professionalism and the critical interview technique were used to help generate SJT item content.^21^ The response format was adopted from that used by the previously validated University Clinical Aptitude Test (UCAT)^22^ SJT. This SJT has two types of item: those that ask candidates to rate the *appropriateness* of a predicted behaviour; and those that ask the test-taker to rate the relative *importance* of an element in the scenario to whether the behaviour depicted was professional or not. The scores from this SJT have been shown to predict third-party ratings of relevant interpersonal functioning. Moreover, the response format of the UCAT SJT seemed a good fit for the construct under evaluation. That is, when testing procedural knowledge of professionalism in mental health settings, it seemed relevant that respondents were able to judge the *appropriateness* of depicted behaviours. Furthermore, it was pertinent that test-takers could identify the elements in a scenario that influenced the professionalism or morality of a depicted behaviour. Following initial item development, the content was reviewed for clarity and pertinence by subject matter experts (SMEs), which included patients, carers and mental health clinicians. The SMEs provided a provisional scoring rubric for each item. Participating SMEs were offered a £30 gift voucher to recompense them for the time they spent on the study.

**Fig. 1:**
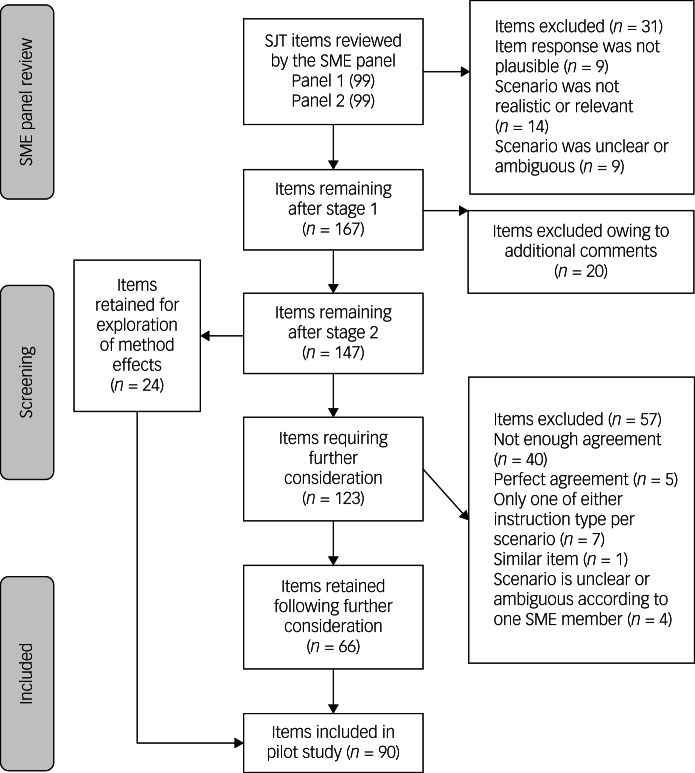
Flow diagram depicting the item selection process. Note: one item was marked as both ‘not plausible’ and ‘not realistic’ during stage 1; hence, the total of these values is 32, yet only 31 items were omitted. SME, subject matter expert.

Corrected Krippendorff's alpha was used, alongside other methods, to calculate the level of agreement among SMEs and guide the shortlisting of items.^23^ The feedback provided by SMEs resulted in a pool of 90 SJT items (Fig. 1). It is worth noting that SMEs had experience as either a patient, carer or employee of mental health and/or learning disability services and had also participated in the focus groups and interviews that contributed to the SJT development. Two of the current authors (L.M.E.A. and P.A.T.) contributed the SME responses having had personal experience of delivering and receiving mental healthcare.

SJT items were mapped to six of ten professional attributes that are required of practitioners working in mental health services: *commitment to professionalism, ability to cope with pressure, effective communication, patient focus, teamwork* and *working with carers*.^19^ Four other professional attributes were not considered when mapping items because they were either implicit to the SJT process (e.g. problem solving) or fell within one of the six attributes named above.^20^ The content themes were distributed evenly across the items of the two forms of the pilot test as part of the ‘blueprinting’ process commonly applied to SJT development.^10,24^ The two forms of the SJT had ten shared items, each having 50 items in total.

### Research design

An observational, cross-sectional, criterion-related validity study was conducted using clinical staff employed by a large mental health service provider in Northern England.

### Participants

All staff in the trust were eligible to participate in the study if they were registered with a clinical professional regulatory body. Nurses and allied health professionals (AHPs) made up the two largest professional groups in the overall study sample, and the findings in relation to these disciplines are reported below. In the UK, the term ‘AHP’ refers to degree-level, professionally autonomous healthcare practitioners who are not doctors, dentists or nurses. With the exception of osteopaths, AHPs are regulated by the Health and Care Professions Council.^25^ It was not possible to do a subgroup analysis for the other disciplines, such as psychiatrists, owing to the relatively small numbers of these individuals participating in the study.

### Procedure

The SJT data were collected between January and October 2020. Participants were randomly assigned one of the two SJT forms (using the RAND function in Excel). The study questionnaire, which included the SJT, requested contact details for their line manager or supervisor and up to three colleagues. These third parties were subsequently contacted by the lead author (L.M.E.A.) for ratings of the participants’ *professionalism* and *effectiveness*. The participants completing the SJT were offered a £10 gift voucher for taking part, and colleagues returning ratings were offered a £5 gift voucher.

### Measures

The electronic study surveys were created using Qualtrics and included an information and consent page, some questions regarding the participants’ demographics and professional characteristics, 50 SJT items, some questions related to the perceived acceptability of the SJT and the personality self-report measure (see below). It was anticipated that the survey would take approximately 30 min to complete.

#### Personality assessment

The Big Five Inventory–2 short form (BFI-2-S) was used to assess the ‘Big Five’ personality traits (extraversion, agreeableness, conscientiousness, negative emotionality and open-mindedness^26^). The BFI-2-S requires participants to rate, on a five-point Likert scale, how well certain statements describe them (e.g. ‘Is outgoing, sociable’, or ‘Worries a lot’).

#### Workplace Behaviours Rating Tool

The Workplace Behaviours Rating Tool was developed to collect feedback from participants’ colleagues, including managers and supervisors. Colleagues were asked to provide two rating scores for participants on a scale from 0 to 100, regarding their perceived *professionalism* and *effectiveness*, using the relative percentile method.^20,27,28^ When asked to provide ratings, colleagues were provided with the following definition of professionalism, which was derived from the findings of an earlier systematic review: ‘Professionalism allows practitioners to make appropriate judgements in times of need, applying critical thinking, reflection and situational judgement.’^17^ As the definition of *effectiveness* is less contentious than that of *professionalism* and may also vary to some extent depending on the role held, no specific definition was provided. The relative percentile method has shown to be a valid approach to capturing third-party ratings of key aspects of workplace performance. In line with previous research regarding the validity of this approach, raters were instructed to provide the score by comparing the ratee with other staff members of the same profession, irrespective of their grade and experience, and provide a specific example of a behaviour observed in the ratee that illustrates the attribute being evaluated.^27^

### Data analysis

Data were anonymised and imported into Stata, where the main data analysis was performed. For analysis purposes, named colleagues were allocated to one of two groups: ‘supervisors’, which incorporated managers, supervisors and professional leads; and colleagues, which included all other individuals. During an initial exploratory analysis, the authors observed that ‘supervisor’ ratings were associated with SJT scores but colleague ratings were not. Indeed, colleagues were observed, on average, to rate participants more positively than supervisors. Thus, only the findings relating to supervisor ratings as the primary outcome are reported here.

A discipline-specific SJT score was obtained for each participant using a ‘dichotomous modal consensus’ scoring approach. In this method, a test-taker is allocated a score of 1 for an item if their response is the most commonly observed response provided by other test-takers in the pilot study; otherwise, a score of 0 is allocated for that item. This acknowledged that that nurses and AHPs have somewhat different roles. Consequently, scores were slightly adjusted depending on the discipline of the test-taker. In order to crudely equate scores across forms and disciplines (AHPs and nurses), the total scores for individuals were standardised as z-scores (mean of 0, s.d. of 1) according to the mean and standard deviations obtained by nurses and AHPs for each form of the SJT. This permitted a pooled analysis. The effectiveness of this equating approach was evaluated by observing the relationship between the standardised dichotomous modal consensus scores and the primary outcomes of interest (supervisor ratings of *professionalism* and *effectiveness*) for the two separate test forms.

#### Selecting items for the final version of the SJT

The findings from the validation study were intended to guide the selection of the most valid items, across content domains, for the final two forms of the test intended to be trialled in practice. Internal consistency reliability of the SJT forms was evaluated using the Kuder Richardson KR20 index.^29^ Internal consistency reliability was not considered when selecting items for the final pool, however, owing to well-documented issues with traditional metrics of reliability in relation to SJTs.^30^ Instead, the final pool of items was prioritised based on the items' criterion-related validity.

## Results

**Fig. 2:**
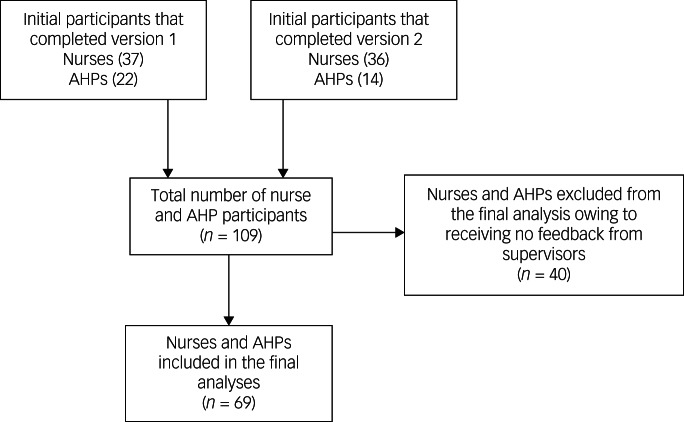
Flow diagram of participation in the validation study. AHP, allied health professional.

The validation study sample consisted of 36 AHPs (33%) and 73 nurses (67%). The AHPs that participated in the study included occupational therapists, dieticians, physiotherapists, and speech and language therapists. The mean age of participants was 41.5 years (s.d. 10.04 years). The sample was predominantly female (84%), and the majority of participants spoke English as their first language (99%). Participants worked across a range of settings and specialties within mental health and learning disability services. Supervisory feedback was received for 69 individuals (Fig. 2). The median scores for *professionalism* and *effectiveness* were 86 (interquartile range [IQR] 75–91) and 81 (IQR 72–91), respectively. The range of ratings observed was 34–100 for *professionalism* and 26–100 for *effectiveness*. Using an independent-samples *t*-test, no significant age difference was observed between participants that did and did not receive supervisor ratings (*P* = 0.94); likewise, using a chi-squared test of independence, no significant difference was observed between participants that did and did not receive supervisor ratings according to their gender (*P* = 0.50). Of those that received supervisor ratings (*n* = 69), the mean total raw scores obtained on the SJT were 33.1 (s.d. 4.01) for form 1 and 34.5 (s.d. 4.44) for form 2. The ranges of raw scores observed were 26–42 for form 1 and 24–42 for form 2. As noted above, the total scores for individuals were standardised as z-scores (with a mean of zero and s.d. of 1) to crudely equate scores across forms and disciplines. The distribution of nurses’ and AHPs’ SJT scores followed a normal distribution according to a quantile–quantile plot.

The correlations between the study variables are displayed in Table 1. As can be seen from the linear regression results shown in Table 2, nurses’ and AHPs’ standardised SJT scores statistically significantly predicted supervisor ratings of both *professionalism* (β = 0.31, *P* = 0.01, *n* = 69) and *effectiveness* (β = 0.32, *P* = 0.01, *n* = 69).

**Table 1:** Means, standard deviations and intercorrelations of study variables – supervisor ratings only (adapted from ref. 20)

Variable	1	2	3	4	5	6	7	8
Standardised SJT score (*N* = 109)								
1. Dichotomous modal consensus score								
Self-ratings on the BFI-2-S (*N* = 109)								
2. S Extraversion	−0.01							
3. S Agreeableness	0.19	0.12						
4. S Conscientiousness	−0.04	0.13	0.24*					
5. S Negative Emotionality	−0.02	−0.26**	−0.25**	−0.27**				
6. S Open-mindedness	0.09	0.24*	−0.01	0.00	0.08			
Job performance (*N* = 69)								
7. Effectiveness	0.30*	0.13	0.36**	0.30*	−0.20	0.10		
8. Professionalism	0.26*	−0.13	0.26*	0.15	−0.02	−0.03	0.80***	
Mean	0	3.52	4.19	3.92	2.52	3.72	80.57	82.92
s.d.	1	0.72	0.54	0.66	0.87	0.69	14.50	12.64

BFI-2-S, Big Five Inventory–2 short form; S, self-report.

Bold indicates significant results (*P < 0.05; **P < 0.01; ***P < 0.001). Calculated using Spearman's rho.

**Table 2:** Results from the regression analyses predicting supervisor ratings of workplace performance from the SJT scores. Note that the standardised coefficients (β) are given in parentheses

Outcome	Coefficient (β)	*P*	Lower 95% CI	Upper 95% CI	*R*^2^ for the model
Univariable results					
Professionalism rating	0.02 (0.31)	< 0.01	0.01	0.04	0.10
Effectiveness rating	0.02 (0.32)	<0.01	0.01	0.04	0.10
Multivariable results (adjusted for personality scores)					
Professionalism rating	13.36 (0.26)	<0.05	1.45	25.28	0.07
Effectiveness rating	10.97 (0.20)	0.10	−2.09	24.03	0.04

SJT, situational judgement test.

### Group differences

No significant difference between females’ and males’ average SJT scores was observed using an independent-samples *t*-test (*P* = 0.55). Group differences according to ethnicity could not be explored, because all but one individual in the nurse and AHP sample identified as being of White ethnicity.

### Face validity

Responses regarding the acceptability of the SJT can be viewed in Table 3; in most cases, the SJT was perceived by nurses and AHPs as relevant to their role, an appropriate difficulty for their grade, suitable for recruitment and fair to all applicants. Having reviewed the feedback in more detail, however, it was apparent that there was a concern that there would be different expectations dependent on the seniority and experience of the responding professional. In addition, one AHP commented that the multiple-choice options were rather restrictive.

‘I'm not sure a multiple choice would work well for getting a full picture of what the person is like. Perhaps if this, combined with a chance for the person to speak openly (not multiple choice) about situations they have experienced may work well.’ (AHP)

**Table 3:** Nurse and AHP perceptions regarding the SJT (*n* = 109)

	Yes (%)	No (%)
Is the test relevant to your role?	96.3	3.7
Is the difficulty of the test appropriate for your grade?	92.7	7.3
Do you think this test would be suitable for the recruitment of staff into the mental health workforce?	92.7	7.3
Do you think the test would be fair to all job applicants, regardless of their profession, gender, age, race and other characteristics?	87.2	12.8

AHP, allied health professional; SJT, situational judgement test.

### Incremental validity

Owing to the limited sample size, only variables that statistically significantly predicted ratings of job performance (*P* < 0.05) in the univariable analysis were entered into a multivariable analysis. This was intended to evaluate the incremental validity of the SJT scores over and above personality self-rating scores. Results of the multivariable analyses are presented in Table 2. Controlling for the potential influence of self-rated *agreeableness* and *conscientiousness*, the SJT scores did not provide statistically significant additional predictive validity with regards to ratings of *effectiveness* (β = 0.20, *P* = 0.10). However, they were observed to do so in relation to predicting supervisor ratings of *professionalism* controlling for self-rated *agreeableness* (β = 0.26, *P* = 0.03). When we selected the final pool of items, they were prioritised based on their relationship with perceived *professionalism*, being ranked by the strength of the observed association of their scores with these supervisor ratings. This resulted in 105 items that could be used to generate a final test score: 44 items in form 1, and 41 items in form 2.

### Reliability

A Kuder Richardson KR20 reliability analysis was carried out on the final items for each SJT form. Alpha coefficients of 0.45 for form 1 and 0.38 for form 2 were observed. Thus, conventional metrics of reliability indicated low to moderate internal reliability consistency. In addition, it is sometimes more appropriate to assess traditional reliability of SJTs, where there are items nested within scenarios, at the ‘testlet’ (i.e. scenario) rather than item level.^31^ Thus, item scores were summed for each scenario that they corresponded to, and the ordinal summed scores were assessed for reliability. This resulted in McDonald's omega values of 0.47 for form 1 and 0.64 for form 2.

We also conducted an analysis to explore whether our approach to equating had resulted in two forms of the test that had similar levels of validity in relation to the outcome of interest. In this regard, the regression coefficients were similar for predicting ratings of both *professionalism* (β = 0.53, *P* = 0.00; β = 0.36, *P* = 0.03) and *effectiveness* (β = 0.45, *P* = 0.01; β = 0.37, *P* = 0.02) for the scores derived from the final items of both form 1 and form 2. This suggests that the equating was at least crudely effective.

## Discussion

This study sought to validate an SJT that was developed to assess procedural knowledge of professionalism in mental health services. The findings from our pilot study provided evidence that the scores validly predicted supervisor ratings of *professionalism* and *effectiveness* in a sample of nurses and AHPs. Moreover, the SJT scores possessed incremental validity in this respect, over and above that provided by self-rated *agreeableness*. The magnitude of the validity coefficients we observed were slightly higher than the mean of 0.26 reported in a general meta-analysis of SJTs for using knowledge-based instructions (i.e. ‘what should you do?’) for personnel selection.^32^ However, they were comparable in magnitude with the mean validity coefficients reported for SJTs used in the context of medical selection.^13^ Importantly, the validity coefficients we observed for our SJT were similar to those reported by a previous meta-analysis of the validity of structured interviews. In this latter case, the mean validity coefficient for structured interviews was cited as 0.31.^33^ SJTs can be implemented at a fraction of the cost of face-to-face interviewing processes and can be delivered at scale, electronically, if required. This capability was especially important during the recent Covid-19 pandemic, when, at times, the risks of face-to-face recruitment processes were seen to outweigh the potential benefits.

It was interesting to note that, in contrast to the supervisor ratings, colleague ratings of *professionalism* were not statistically significantly associated with SJT performance. As mentioned earlier, it is possible that test-takers chose colleagues that would rate them more favourably, especially given the financial incentive for taking part. That is, there was some choice in who provided this rating, whereas this would not have been the case regarding supervisor or line manager ratings. Thus, we excluded colleague ratings in subsequent analyses.

### Strengths and limitations

The participants in our pilot study were already clinicians working in mental health services. This sample would have inevitably restricted the range of both predictor (SJT scores) and outcomes (supervisor ratings) in the data. This would have attenuated the observed validity coefficients to some extent. That is, had the SJT been piloted on an applicant sample, more variation may have been observed among SJT scores, which in turn would have influenced the reliability and validity coefficients observed.^34^ Range restriction is a common challenge with validation studies.^35^ In the current study, both the selection assessment and the outcomes could only be observed, which prohibited the researchers from making the usual mathematical adjustments for direct and indirect restriction of range in these contexts.^36–38^ Despite this, meaningful and statistically significant correlations were observed, providing evidence for the validity of the SJT scores in this context.

There are both advantages and disadvantages of using the dichotomous modal consensus scoring approach. First, using dichotomous modal consensus scoring makes the modelling of responses more parsimonious. Dichotomous scoring systems tend to make SJT response patterns more unidimensional compared with polytomous scoring. This is especially helpful when attempting to equate several forms of the same test. Also, intergroup bias (for example, according to ethnicity) can, in theory, be amplified by using polytomous scoring systems. This is because of well-documented tendencies for certain ethnic groups to show extreme response styles when answering questionnaires.^39^ This can introduce undesirable bias into tests. The main disadvantage of a dichotomous scoring systems is the potential information loss. That is, moving to dichotomous scoring systems will inevitably reduce, albeit modestly in most cases, test information. This will therefore adversely affect the ability of the test to discriminate between candidates at different levels of the relevant trait(s). However, this may be optimally traded off by the potential advantages outlined earlier.

Ideally, actual clinical practice or patient outcomes would have been captured as the criterion-related outcome measure. However, actual patient outcomes are challenging to capture in mental health services, and there are confounding factors, such as team-level effects at work. Thus, we used the traditional approach of capturing supervisor ratings, using the relative percentile ranking method, in an attempt to mitigate rater effects. Rater behaviour can be psychometrically understood, and at times adjusted for, using either generalisability (G) theory^40^ or the many-facet Rasch model.^41^ However, this would require a more extensive, linked data structure than was available in this case. This may be worth considering when designing future research involving third-party ratings of in-job performance.

Responses to SJT items, in this context, tend to be multidimensional. More specifically, they are best described as ‘fuzzy unidimensional’^42^ or ‘essentially unidimensional’.^43^ This involves having one main, general latent variable (factor) that items load on, with a number of smaller factors that may also cross-load on the main factor, or other minor factors. This lack of unidimensionality causes challenges when evaluating the reliability of SJTs.^30^ Nevertheless, many authors continue to report reliability using classical metrics (such as Cronbach's alpha coefficient) when describing findings from SJT-related studies. For transparency, in the present study we assessed and reported on the internal consistency values of the SJT forms, which demonstrated relatively low internal consistency. Relatively low internal consistency values are fairly typical of SJTs used in this context. For example, a meta-analytic study found that the average observed reliability of SJTs used in personnel selection (mean of 0.61, s.d. of 0.20) was much lower than that typically observed in high-stakes assessments (usually >0.80).^44^ Therefore, ‘alternate forms’ and test–retest reliability have been suggested as more appropriate metrics of SJT reliability.^32^ We plan to assess the final SJT in practice in this respect in the near future. Moreover, in this context, criterion-related validity values for SJTs tend to be considered as the most important psychometric property of these assessments.

Despite a general lack of validity, personality tests are still commonly used in personnel selection.^45^ Thus, controlling for this factor was appropriate. However, academic achievement and cognitive ability are also sometimes tested for as part of selection, particularly for senior mental health roles. Thus, ideally these factors should also have been controlled for when evaluating the incremental validity of the SJT scores. Previous research has tended to indicate that SJTs, at least for medical selection, tend to show incremental predictive ability beyond academic or cognitive ability.^13^

### Implications for policy and practice

There have been many instances where staff have failed to provide adequate care to patients. The current SJT would flag applicants that provide ‘unusual’ responses to the items in the assessment (i.e. those that would be unusual for that professional discipline). Such unexpected responses could then be explored in a face-to-face interview. It is hoped that in doing so, more suitable candidates for mental health services would be selected that had appropriate knowledge for the role. In some selection contexts, SJTs are used to ‘screen out’ low-scoring candidates at an early recruitment stage. However, in the absence of ‘post-marketing’ evidence of validity of the SJT when used in a high-stakes setting, the authors suggest that the scores should not be used as a hard ‘rejection’ or ‘acceptance’ rule at this point. Individual employing organisations would have to decide on the most practical and potentially effective way of using the SJT within their recruitment process. Within this, test security would have to be considered. This may include online or in-person proctoring and other precautions to prevent cheating or test content leakage. It is worth noting that SJTs have been used successfully for training and development previously.^24,46^ Thus, it is possible that items from our pilot test that were not selected for the final SJT assessment could be used as part of staff training.

Owing to the nature of our staff sample, it was not possible to evaluate the validity of the SJT in other professional disciplines in mental health, for example, clinical psychology or psychiatry. However, given the differing training, perspectives and nature of clinical work across disciplines, it is likely that specific SJTs would have to be developed for selection or training purposes in different professional groups. Moreover, the scoring system would also have to be calibrated for specific disciplines of practitioners.

### Recommendations for further research

This study identified 105 SJT items that should be used to generate a final test score. It is thus important that a longitudinal evaluation is conducted to establish the validity of the final SJT version in actual practice. Such an evaluation could assess the cross-sectional relationship between SJT scores and interview performance ratings, as well as more distal outcomes such as retention in the workforce or, given a large enough sample, patient feedback on quality and complaints. In this regard, SJT scores have previously been shown to predict future disciplinary action among UK medical students.^47^ A longitudinal study would also be an opportunity to assess test–retest reliability, given the limitations of traditional reliability estimates for SJTs.

Only a small number of participants identified as being from a minority ethnic group. Future studies could explore group differences in SJT scores for this population, given the implications for equality and diversity. In this respect, the evidence relating to SJTs is mixed, although there are some indications that SJTs are less sensitive to socioeconomic factors than other selection assessments such as cognitive ability tests.^48^ There is currently no published research on the potential adverse impact of SJTs on individuals affected by neurodevelopmental conditions such as autism spectrum conditions. We did not collect data regarding participants’ neurodiversity. Relative cognitive inflexibility is a core component of autism spectrum conditions. Consequently, we suspect that such individuals may be less disadvantaged by a dichotomous compared with a polytomous SJT scoring systems. That is, such individuals may find it harder to make more subtle distinctions between response options. Future research could explore the impact of scoring systems on individuals who may be affected by neurodivergence.

Traditionally, SJT scoring systems rely on SMEs, which rarely include carers and patients. However, after exploring a number of commonly used SJT scoring systems, it was noted that the dichotomous modal consensus scoring system demonstrated the most validity. It was also relatively easily to implement and automate scoring using a binary rather than a polytomous system. Nevertheless, it should be highlighted that consensus scoring relies on the ‘wisdom of crowds’. In this context, deriving consensus from a group of fairly experienced, professionally registered mental health clinicians may be a relatively safe scoring strategy. It does, however, risk placing SJT scoring in a professional ‘hall of mirrors’. That is, professionals may agree among themselves what the most appropriate or effective response to a particular interpersonal situation might be, but would carers or mental health patients agree? Moreover, if the criterion-related outcome is supervisor ratings, as is generally the case in SJT validation studies, this is yet another reflective surface in the professional ‘hall of mirrors’. Thus, future studies might wish to be more ambitious and inclusive in capturing the voice and views of patients and carers, not just in the design of an SJT but in the scoring and validation process itself.

This study demonstrates that an SJT that is capable of being delivered digitally and at scale is a potentially valid tool that can enhance and support VBR into clinical roles in mental health services. Indeed, the validity may be comparable with that observed for structured interviews. It is this validity and cost-effectiveness that has led to the popularity of this personnel selection approach in many fields, including medicine. Now that this method is available to mental health services, it is hoped that it will lead to more of the right individuals, with an understanding of how the desired values for professional, compassionate and person-centred care should be exhibited in practice, caring for our most vulnerable patients.

## Data Availability

The data that support the findings of this study are available from the corresponding author, L.M.E.A., upon reasonable request.
